# Deletion of TSPO Resulted in Change of Metabolomic Profile in Retinal Pigment Epithelial Cells

**DOI:** 10.3390/ijms20061387

**Published:** 2019-03-19

**Authors:** Abdulwahab Alamri, Lincoln Biswas, David G. Watson, Xinhua Shu

**Affiliations:** 1 Department of Pharmacology, College of Pharmacy Sciences, University of Hail, Hail 55476, Saudi Arabia; ph.whab@gmail.com; 2 Strathclyde Institute of Pharmacy and Biomedical Science, University of Strathclyde, Glasgow G4 0RE, UK; 3 Department of Biological and Biomedical Sciences, Glasgow Caledonian University, Glasgow G4 0BA, UK; Lincoln.Biswas@gcu.ac.uk; 4 Department of Vision Science, Glasgow Caledonian University, Glasgow G4 0BA, UK

**Keywords:** TSPO, metabolites, retinal pigment epithelial cells, age related macular degeneration

## Abstract

Age-related macular degeneration is the main cause of vision loss in the aged population worldwide. Drusen, extracellular lesions formed underneath the retinal pigment epithelial (RPE) cells, are a clinical feature of AMD and associated with AMD progression. RPE cells support photoreceptor function by providing nutrition, phagocytosing outer segments and removing metabolic waste. Dysfunction and death of RPE cells are early features of AMD. The translocator protein, TSPO, plays an important role in RPE cholesterol efflux and loss of TSPO results in increased intracellular lipid accumulation and reactive oxygen species (ROS) production. This study aimed to investigate the impact of TSPO knockout on RPE cellular metabolism by identifying the metabolic differences between wildtype and knockout RPE cells, with or without treatment with oxidized low density lipoprotein (oxLDL). Using liquid chromatography mass spectrometry (LC/MS), we differentiated several metabolic pathways among wildtype and knockout cells. Lipids amongst other intracellular metabolites were the most influenced by loss of TSPO and/or oxLDL treatment. Glucose, amino acid and nucleotide metabolism was also affected. TSPO deletion led to up-regulation of fatty acids and glycerophospholipids, which in turn possibly affected the cell membrane fluidity and stability. Higher levels of glutathione disulphide (GSSG) were found in *TSPO* knockout RPE cells, suggesting TSPO regulates mitochondrial-mediated oxidative stress. These data provide biochemical insights into TSPO-associated function in RPE cells and may shed light on disease mechanisms in AMD.

## 1. Introduction

Age-related macular degeneration (AMD) is a cumulative chronic disorder of the central retina and a principal cause of blindness in industrialized countries [1]. Drusen are abnormal extracellular deposits between the retinal pigment epithelium (RPE) and Bruch’s membrane and represent hallmarks of AMD. The sizes of drusen are suggested to be associated with the severity of AMD: early AMD is characterized by medium-sized drusen without pigmentary abnormalities, while intermediate AMD contains medium-sized drusen and pigmentary abnormalities or large-sized drusen with or without pigmentary abnormalities [2]. Drusen contain heterogeneous materials, including carbohydrates, lipids and proteins. Lipids, dominated by esterified cholesterol and phosphatidylcholines, are abundant in drusen [3]. Deposition of lipids affects Bruch’s membrane and leads to blockage of fluid exchange and macromolecular permeability between the choroid and the RPE [3]. Accumulated lipids and lipoproteins are susceptible to oxidation due to the high oxidative stress microenvironment. Oxidized lipids such as oxidized phospholipids and oxidized lipoproteins (e.g., oxidized LDL) have been reported in drusen, which can act as a trigger for proinflammatory events, including complement activation, in the pathogenesis of AMD [4,5,6] Additionally, genome-wide association studies have demonstrated that genetic variants in lipid metabolism and transport genes, including hepatic lipase (LIPC), cholesterol ester transferase (CETP), apolipoprotein E (APOE) and ABC binding cassettes A1 (ABCA1), are associated with an increased risk of AMD [7,8,9]. These data suggest dysregulation of lipid metabolism and transport contributes to the early pathogenesis of AMD.

RPE cells support photoreceptor cells through particular functions, such as the supply of nutrients and the removal of waste, in addition to phagocytosis and handing of the photoreceptor outer segment shedding process [3]. Proteins associated with cholesterol synthesis, metabolism and transport have been reported to be expressed in the RPE. RPE cells are responsible for cholesterol efflux and uptake which is eventually processed and delivered as HDL-like particles to HDL receptors on the photoreceptor cells; RPE cells also transport cholesterol to the sub-RPE space for clearance through the choriocapillaries [10,11,12]. Therefore, RPE cells act as the main site of cholesterol and other lipid transportation through different proteins to the inner and outer sides of the retina. The existence of apolipoproteins, cholesterol and cholesteryl ester deposits beneath the RPE cells has been reported in AMD patients, linking irregular cholesterol transport to the progression of disease [3].

The translocator protein (TSPO), an 18 kDa protein localized to the outer mitochondrial membrane, is one of the complex proteins involved in mitochondrial cholesterol trafficking and is believed to mediate several other functions including neuroinflammation, mitochondrial homeostasis and apoptosis [13]. In previous work, TSPO was found to be strongly expressed in human and mouse RPE cells and participated in mediating cholesterol efflux from RPE cells [11]. The study also found significant increases in cholesterol uptake and accumulation in *TSPO^−/−^* RPE compared to wildtype cells, confirming that TSPO deletion impaired cholesterol efflux and lipid transport in RPE cells.

Recently metabolomics has been applied to the investigation of disease mechanisms and the identification of biomarkers distinguishing patients from healthy subjects for different eye diseases including AMD [14]. Osborn et al. carried out metabolomic analysis of plasma samples from control and neovascular AMD (NVAMD) using liquid chromatography mass spectrometry (LC/MS) and identified 94 metabolites that were significantly different between the controls and NVAMD. Further analysis of 40 metabolites demonstrated that peptide and amino acid metabolism was most significantly changed in NVAMD plasma samples [15]. Two other groups also used LC/MS to metabolomically characterize NVAMD and control plasma samples [16,17]. Luo et al. found ten metabolites were significantly different between the control group and the NVAMD group and that predominant changes occurred in the amino acid metabolic pathway [16]. Mitchell et al. identified 159 metabolites in the NVAMD group that were significantly different from the controls and found that the carnitine shuttle pathway was notably changed in NVAMD patients [17]. Metabolomic analysis of plasma samples from AMD patients at early, intermediate and late stages using nuclear magnetic resonance spectroscopy only detected small changes in the levels of some amino acids, organic acids, lipid moieties and proteins in AMD patients [18]. Further metabolomic analysis of plasma samples from 30 controls and 90 AMD patients (30 in early stage, 30 in intermediate stage and 30 in late stage) by LC/MS identified 87 metabolites that were significantly different between AMD patients and controls, with the predominant change occurring in metabolites that functioned in lipid metabolism [19]. Recently Li et al. characterized serum lipidomics in polypoidal choroidal vasculopathy (PCV), a subtype of NVAMD, using LC/MS. They identified 41 metabolites that differed significantly between the controls and the PCV patients [20]. Among the identified metabolites, platelet-activating factors (PAF) were markedly increased in PCV patients, suggesting PAF may play an important role in the pathogenesis of NVAMD.

In the current study, metabolomic analyses were employed on wildtype and TSPO^−/−^ RPE cells treated with or without oxidized low density lipoprotein (oxLDL) to detect key metabolic changes associated with loss of TSPO and oxidative stress induced by oxLDL.

## 2. Results

### 2.1. Principle Component Analysis (PCA) and Orthogonal Projections to Latent Structures Discriminant Analysis (OPLS-DA)

PCA aims to classify the samples into groups of similar characteristics but different from those in other groups while the model is unsupervised and the samples are plotted with no information given about the group classes. It demonstrates the possible presence of outliers, groups, similarities and other data patterns. When the PCA score plot illustrates a clustering pattern, identifying the differences between the groups by performing a PCA classification or OPLS-DA is recommended.

Samples of wildtype and TSPO knockout cells, with or without oxLDL treatment, were prepared and injected into the LC-MS. The data was then extracted and processed to detect metabolites that were different between wildtype and TSPO knockout cells, or between oxLDL treated and untreated cells. Initially, system stability was examined by running pooled (quality control) samples throughout the experiment after every three samples. Pooled samples were clustered together in the PCA plot (Figures S1 and S2), indicating reasonable system stability and high precision. Moreover, relative standard deviation (RSD) values were calculated based on total intensities in each of the pooled samples and total RSD of 19.58% was obtained. Metabolites with RSD values higher than 30% across the pooled samples were excluded. Clustering of QC samples (P1, 2, 3, 4 and 5) reflected good precision and the extra assessment of RSD was used to ensure that differences between samples were based on the intervention and were not due to instrumental factors.

Comparative metabolomics was employed by applying metabolite extraction and identification workflow to categorize metabolic alterations due to treating wildtype and *TSPO^−/−^* cells with oxLDL and comparing them to the control of each type. The study was carried out in duplicate and samples were used for each group (*n* = 3) and consistent results were considered. Data were filtered according to a CV-ANOVA test of each comparison and the relative standard deviation (RSD) for metabolites obtained from pooled samples. Common markers distinguishing the groups are listed based on the *t* test reading. Fold change ratios were calculated for each metabolite for each comparison. Metabolites with significant changes in the comparisons are listed in Table S1.

**Figure 1:**
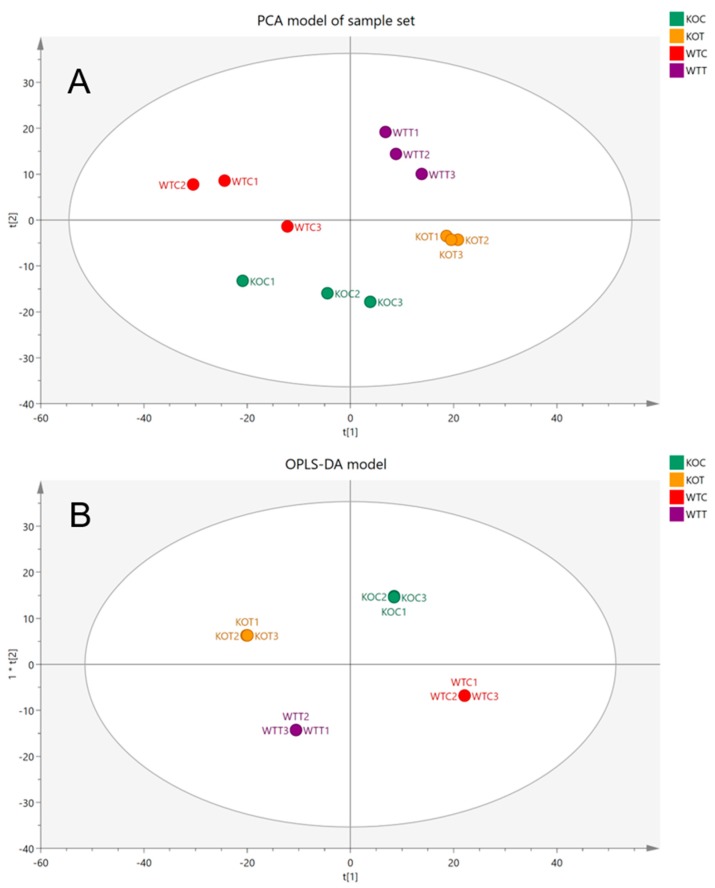
(**A**) Principal components analysis (PCA) plots of four different groups: WTC, wildtype control cells without treatment of oxLDL; WTT, wildtype cells treated with oxLDL; KOC, *TSPO* knockout control cells without treatment of oxLDL; KOT, TSPO knockout cells treated with oxLDL. (**B**) OPLS-DA score plots the samples according to their classification colored based on their group and the reading of 1106 putative metabolites.

The wildtype cells without oxLDL treatment (WTC), wildtype cells with oxLDL treatment (WTT), *TSPO* knockout cells without oxLDL treatment (KOC) and *TSPO* knockout cells with oxLDL treatment (KOT) were evaluated by using a PCA model. Scot plots demonstrated clear separation and clustering of each group showing the difference in metabolic profiles between different groups (Figure 1A). In addition, an OPLS-DA supervised model was used to highlight the differences between groups and to examine the effect of intervention (Figure 1B). The model evaluation parameters, *R*^2^ = 0.99 and *Q*^2^ = 0.71, indicated that the OPLSDA model was reliable and valid. The current model that was built, based on the total reading of 1106 metabolites, showed a clear separation between the four different groups. However, we could not detect the discrimination features that separated each group from others in a model of four groups. Therefore, subsequently, new models were built based on the total reading of 1106 metabolites to compare each two different groups in separate models. The new built models compared the oxLDL-treated *TSPO* knockout (KOT) group to the oxLDL-treated wildtype (WTT) group, the untreated *TSPO* knockout (KOC) group to the untreated wildtype (WTC) group, the oxLDL-treated TSPO knockout (KOT) group to the untreated *TSPO* knockout (KOC) group, and the oxLDL-treated wildtype (WTT) group to the untreated wildtype (WTC) group. This assisted with detection of statistically significant metabolites that contribute to the discrimination between two groups. Outcomes of these comparisons provide better understanding about the differences between wildtype and *TSPO* knockout RPE cells in terms of the metabolic modifications that occur due to oxLDL treatment and TSPO deletion.

### 2.2. The Lipid Pathway Was Most Affected

**Figure 2:**
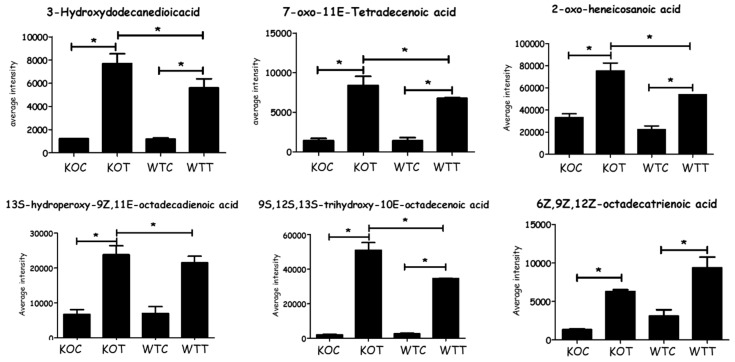
Significant changes of some fatty acids as a consequence of oxLDL treatment in different groups. * Comparison indicates that difference is significant with *p* < 0.05. KOT: *TSPO* knockout cells with the treatment of oxLDL; KOC: *TSPO* knockout cells without the treatment of oxLDL; WTT: Wildtype cells with the treatment of oxLDL; WTC: Wildtype cells without the treatment of oxLDL. Error bar is the standard error of the mean (SEM).

Based on all the identified metabolites, lipids were the most significantly influenced metabolites in response to the stress raised due to TSPO deletion and/or treatment with oxLDL, representing 51% of total affected metabolites, followed by amino acid metabolism pathways at 19%, and the nucleotide metabolism pathway was the least affected at 8% (Figure S3). There were no significant differences in carnitine levels between WTC and KOC cells. OxLDL treatment greatly increased the levels of long chain acyl carnitines in both KOC and WTC cells (Table S1). While the levels of oxidised fatty acids in KOC and WTC cells were similar, there was a very large increase in the levels of some fatty acids and particularly oxidized fatty acids (Figure 2), possibly resulting from oxLDL treatment, and this might be attributed to the oxLDL, which could be contributing these components to the cell extracts.

**Figure 3:**
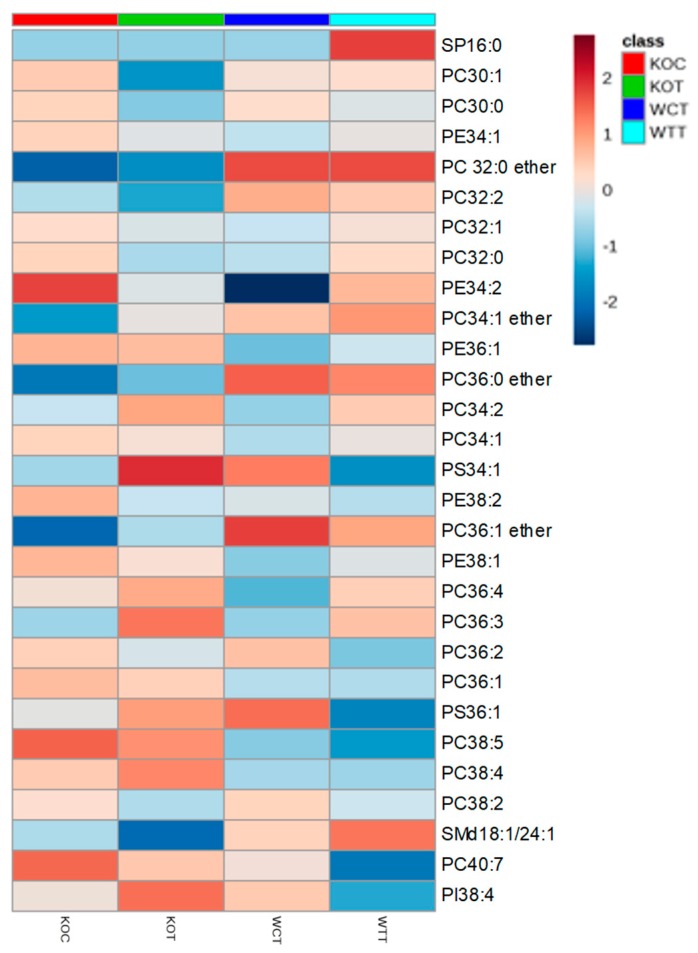
The relative intensity of the 30 most abundant lipids extracted from different groups of cells. Color represents the average intensity of group samples for each certain metabolite. Color scale of highest value was colored in dark green, the midpoint value colored in yellow and dark red for the lowest value. KOT: *TSPO* knockout cells with the treatment of oxLDL; KOC: *TSPO* knockout cells without the treatment of oxLDL; WTT: Wildtype cells with the treatment of oxLDL; WTC: Wildtype cells without the treatment of oxLDL.

Lipids were strongly affected by the treatments. There were differences in the phospholipids between WTC and KOC cells. Figure 3 indicates that four ether lipids were more abundant in the wildtype cells in comparison to *TSPO^−/−^* cells. There were marked effects on the range of phospholipids resulting from oxLDL treatment for both wildtype and *TSPO^−/−^* cells. Many of these lipids were of low abundance, such as the phosphotidyl glycerol lipid PG 44:12 and PC lipids 40:7 and 42:7 (Table S1), but some high abundance lipids were also affected, such as PC 34:2 (Figure 3). Figure 3 shows a heatmap for the 30 most abundant lipids extracted from the cells. The second most abundant lipid, a PC with 36 carbons in the acyl chains and 2 units of unsaturation, decreased after treatment, as did a PC lipid with 32 carbon atoms and 0 units of unsaturation, while an abundant lipid, PC34:2, increased after treatment. As these lipids are abundant, this may indicate some major re-modelling of the cell membrane in response to oxidative stress. There are interesting differences in the less abundant lipids, with several ether lipids being markedly increased and some lipids with highly unsaturated chains being decreased, which is possibly indicative of oxidative stress, while others were increased (Table S1). In particular, a glycolipid with 44 carbon atoms in its acyl chains and 12 units of unsaturation was decreased in the treated samples. It is likely that this lipid is substituted with two docosahexenoic acid chains and such lipids are known to serve an important function in the retina [21].

### 2.3. Metabolic Changes in Glucose Metabolism

**Figure 4:**
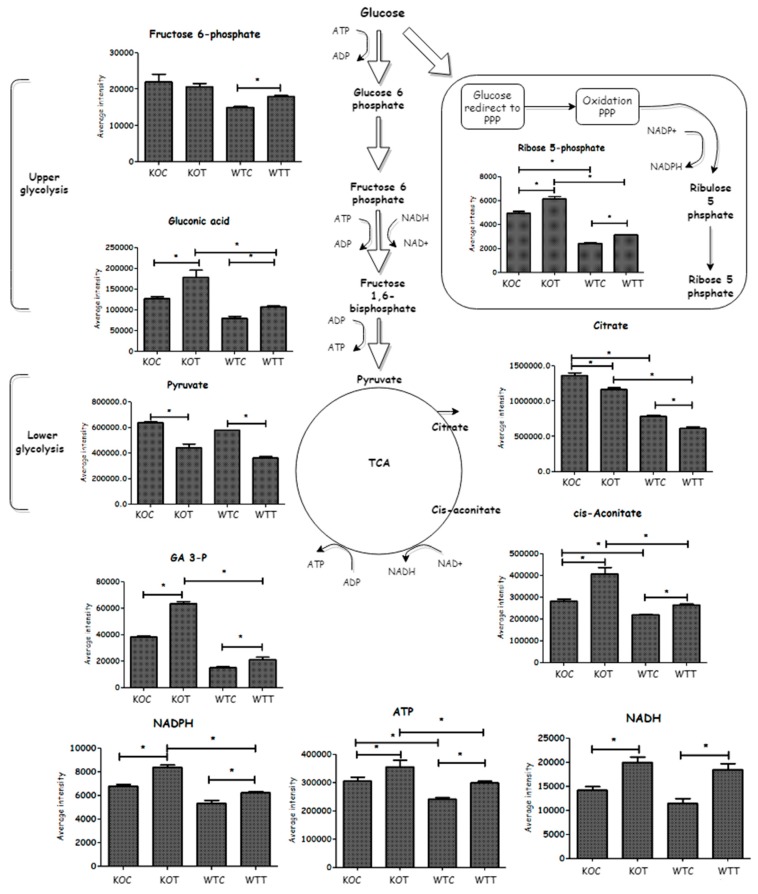
Schematic representation of glycolysis, pentose phosphate pathways (PPP) and TCA cycle. This clarifies how glycolysis was induced by treatment that increases ATP generation. Included column figures demonstrate average metabolite intensity in different groups. Glucose flux into oxidation PPP reflects the impact of *TSPO* knockout and oxLDL treatment in RPE cells. NADPH provides insight into the redox status of cells, while various factors play an important role in its synthesis and consumption. * Indicates that difference is significant with corrected *p* < 0.05. KOT: *TSPO* knockout cells with the treatment of oxLDL; KOC: *TSPO* knockout cells without the treatment of oxLDL; WTT: Wildtype cells with the treatment of oxLDL; WTC: Wildtype cells without the treatment of oxLDL. Error bar is the standard error of the mean (SEM).

Several glycolytic metabolites were elevated in *TSPO^−/−^* cells compared to wildtype cells. Deletion of TSPO influenced metabolites of the pentose phosphate pathway (PPP) because *TSPO^−/−^* cells demonstrated higher levels of ribose 5-phosphate and gluconate 6-phosphate than wildtype. An increased level of the TCA cycle component, cis-aconitate, was observed in KOC compared to WTC conditions, while citrate was lower in wildtype cells than that of *TSPO* knockout cells. NADH and ATP levels were higher in the KOC cells, possibly indicating a faster metabolic rate in these cells. This suggests that TSPO deletion mainly causes increased glucose metabolism, which was obvious when KOC was compared to WTC (Figure 4).

Glucose metabolism (glycolysis, TCA cycle and PPP) was also affected by oxLDL treatment (Figure 4). Glycolysis metabolites, fructose 6-phosphate and gluconic acid, were significantly increased in WTT compared to WTC. Fructose 6-phosphate on the contrary was not notably changed in KOT when compared to that of KOC, but gluconic acid was significantly increased in KOT compared to KOC. Glyceraldehyde 3-phosphate (GA3-P) accumulated due to oxLDL treatment only in knockout but not wildtype cells. OxLDL treatment decreased pyruvate in both wildtype and *TSPO* knockout cells. The PPP metabolites, ribose 5-phosphate and gluconate 6-phosphate, were slightly increased when wildtype and *TSPO* knockout cells were treated by oxLDL in comparison to untreated controls. Thus both wildtype and knockout cell types exposed to oxLDL showed an ability to compensate for oxidative stress by producing increased levels of NADPH. The TCA metabolites (cis-aconitate and succinate) were also induced by oxLDL treatment and this was accompanied by an increase in the generation of NADH, ATP and creatine phosphate, which forms in order to export ATP, formed from the terminal respiratory chain, out of the mitochondria. However, the other TCA component, citrate, was significantly decreased in oxLDL treated cells when compared to untreated cells and this could be explained by citrate also being utilised to produce NADPH, which is formed via the action of isocitrate dehydrogenase. Thus, both cell types respond to oxidative stress by up-regulating energy production and oxidative defense. Therefore, although KOC cells suffer from underlying oxidative stress, their ability to mount a defense against additional oxidative stress is not impaired.

### 2.4. Metabolic Changes in Amino Acid Metabolism

**Figure 5:**
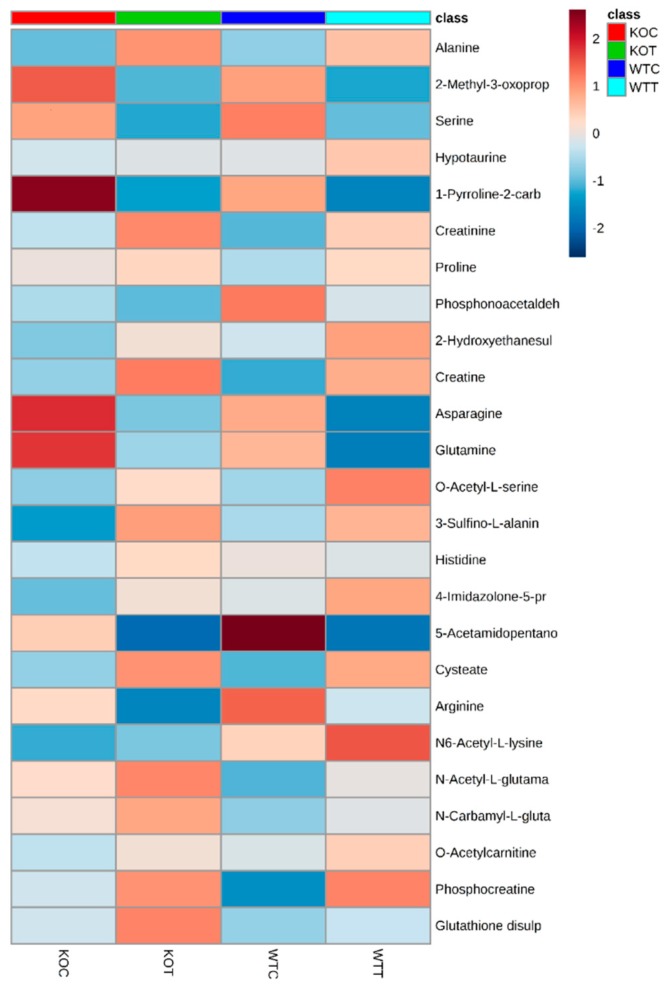
Heatmap of amino acid metabolites that were significantly altered due to oxLDL treatment. It also shows how metabolites were influenced in different groups. Color scale of the highest value is colored in dark green, the midpoint value is colored in yellow and dark red for the lowest value. KOT: *TSPO* knockout cells with the treatment of oxLDL; KOC: *TSPO* knockout cells without the treatment of oxLDL; WTT: Wildtype cells with the treatment of oxLDL; WTC: Wildtype cells without the treatment of oxLDL.

**Figure 6:**
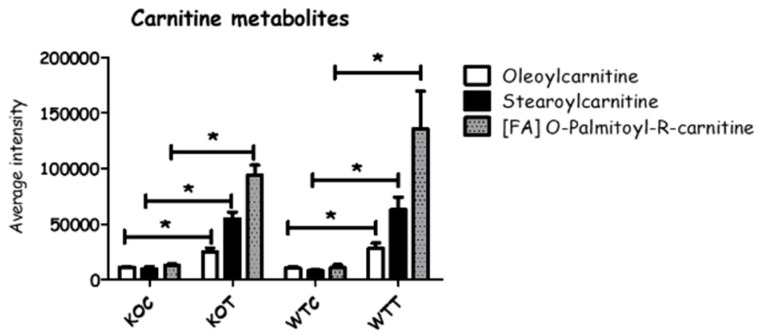
Some carnitine metabolites were changed between different groups. KOT: *TSPO* knockout cells with the treatment of oxLDL; KOC: *TSPO* knockout cells without the treatment of oxLDL; WTT: Wildtype cells with the treatment of oxLDL; WTC: Wildtype cells without the treatment of oxLDL. * Indicates that difference is significant with corrected *p* < 0.05. Error bar is the standard error of the mean (SEM).

When comparing WTC and KOC, quite a number of amino acids and amino acid metabolites were altered. In the arginine pathway, there was a slight decrease in arginine, which may reflect an increased requirement for creatine and creatine phosphate in the KOC cells; both proline and *N*-acetylglutamate, which are potential precusors of arginine, were elevated in KOC. Taurine and one of its precursors, cysteic acid, were elevated in KOC compared to WTC; taurine is an important antioxidant. Amino acid metabolism was disturbed by oxLDL treatment in both wildtype and *TSPO* knockout cells where some metabolites were up-regulated, and some others were down-regulated (Figure 5 and Table S1). Among 27 metabolites detected, 5 metabolites decreased, 16 increased and 6 were not changed when comparing KOT to KOC, and almost similar alterations were observed in WTT compared to WTC. On the other hand, 12 amino acid metabolites were found to be increased in KOT compared to WTT, and two metabolites were significantly decreased. Creatine, cysteate, 3-sulfino-l-alanine, phosphocreatine, alanine, creatinine and *N*-Acetyl-l-glutamate were the most influenced metabolites by oxLDL in wildtype and *TSPO* knockout cells, although they were induced in KOT and KOC more so than WTT and WTC. Serine and arginine were significantly decreased by oxLDL in both wildtype and knockout cells, and were also reduced in knockout cells compared to wildtype cells with or without oxLDL treatment. Carnitine is synthesized from lysine side chains and has a role in transporting the long chain fatty acid into mitochondria. Carnitine metabolites were markedly increased in oxLDL treated wildtype and *TSPO* knockout cells compared to untreated cells (Figure 6).

### 2.5. Metabolic Changes in Nucleotide Metabolism

**Figure 7:**
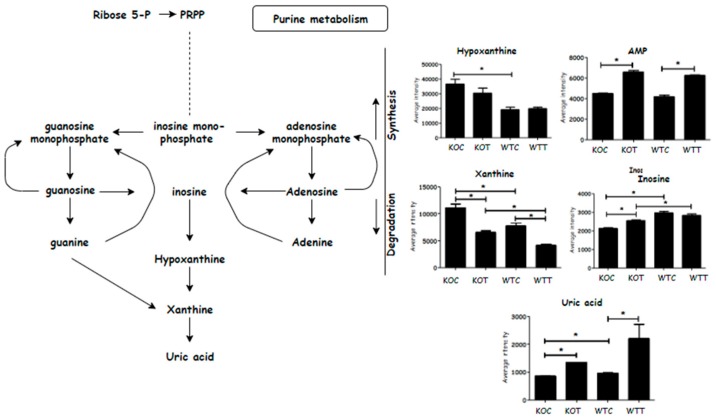
Purine substrates that were altered due to oxLDL treatment. AMP: adenosine monophosphate; PRPP: Phosphoribosyl pyrophosphate. KOT: *TSPO* knockout cells with the treatment of oxLDL; KOC: *TSPO* knockout cells without the treatment of oxLDL; WTT: Wildtype cells with the treatment of oxLDL; WTC: Wildtype cells without the treatment of oxLDL. * Indicates that difference is significant with corrected *p* < 0.05. Error bar is the standard error of the mean (SEM).

Metabolic profiling of some purine metabolites demonstrated that adeno monophosphate (AMP) was induced by oxLDL in both wildtype and *TSPO* knockout cells (Figure 7). It also showed slight increase of inosine in KOT compared to KOC but no difference between WTT and WTC was observed. The levels of hypoxanthine and xanthine were significantly increased in *TSPO* knockout cells compared to wildtype cells; however, hypoxanthine showed no statistical difference between WTT and WTC, or between KOT and KOC. Xanthine, on the other hand, was significantly decreased when either wildtype or knockout cells were treated with oxLDL. Uric acid was notably increased in oxLDL treated cells compared to untreated cells. It was also higher in WTC than KOC (Figure 7).

Pyrimidine pathway components, such as thymine and 5,6-dihydrothymine, were significantly influenced by oxLDL treatment. They showed higher levels among KOT and KOC than WTT and WTC, respectively. Thymine is well known to be broken down into 3-aminoisobutyric acid in the last step of pyrimidine degradation, which eventually enters the TCA cycle. However, 3-aminoisobutyrate was not affected by oxLDL or by TSPO deletion, while no difference was observed between any of the comparisons. Cytosine and 5-methylcytosine were down-regulated by oxLDL treatment and also decreased among knockout cells compared to wildtype cells, with or without oxLDL treatment (Table S1).

### 2.6. Increased Oxidative Stress in OxLDL-Treated Cells

**Figure 8:**
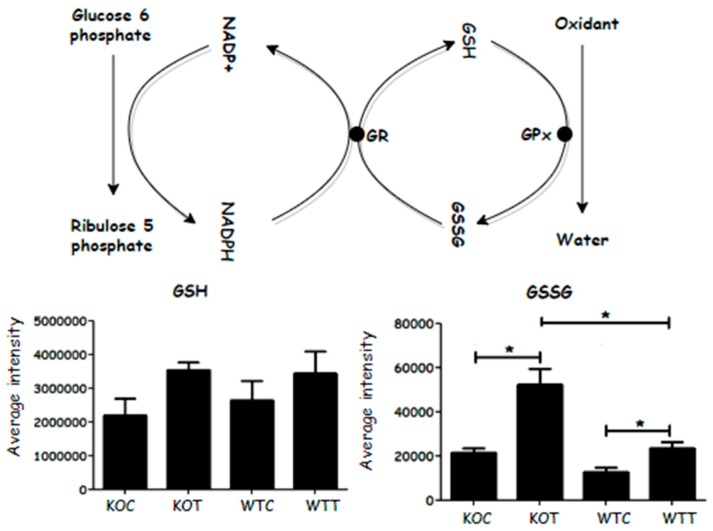
Oxidized glutathione (GSSG) converts to its reduced form in the presence of NADPH, which donates a proton and is converted to its oxidized form, NADP+. GSSG increased significantly when knockout cells were treated by oxLDL. GSH: glutathione; GSSG: glutathione disulphide; GR: glutathione reductase; GPx: glutathione peroxidase. KOT: *TSPO* knockout cells with the treatment of oxLDL; KOC: *TSPO* knockout cells without the treatment of oxLDL; WTT: Wildtype cells with the treatment of oxLDL; WTC: Wildtype cells without the treatment of oxLDL. * Indicates that difference is significant with *p* < 0.05. Error bar is the standard error of the mean (SEM).

TSPO deletion and oxLDL treatment have been reported to induce increased reactive oxygen stress (ROS) in RPE cells [11]. Reduced glutathione is converted to form oxidized glutathione disulphide (GSSG) in the presence of hydrogen peroxide and glutathione peroxidase. A higher GSSG level was detected in oxLDL-treated cells when compared to untreated cells; *TSPO* knockout cells produced more GSSG compared to wildtype cells, with or without oxLDL treatment. NADPH is required to reduce GSSG back to GSH in the presence of glutathione reductase. The increased level of NADPH was not sufficient to counteract an increase in GSSG in the KOC and KOT cells. However, GSH levels were not significantly affected in the KOC and KOT cells (Figure 8). Therefore, *TSPO* knockout cells generally demonstrated a higher oxidative stress response due to an imbalance of GSSG and GSH.

## 3. Discussion

In this study, a LC/MS metabolomics approach was used to quantitatively investigate metabolite changes in wildtype and *TSPO* knockout RPE cells, with or without oxLDL treatment. The data demonstrate dysregulation of different metabolic pathways affected by loss of TSPO and treatment with oxLDL. Our observations revealed higher intracellular levels of carnitine, fatty acids, glycerophospholipids, eicosanoids, sphingolipids and glutathione homeostasis metabolites when either wildtype or *TSPO* knockout cells were treated with oxLDL compared to untreated cells.

In *TSPO* knockout cells the clearest effect on metabolism in comparison with wildtype was increased oxidative stress, as evidenced by an increased level of GSSG in the cells. Associated with this was an increase in phosphogluconate, a major source of NADPH, which is required to recycle GSSG back into GSH. NADPH levels were also slightly elevated in the knockout cells but GSH levels were unaffected, indicating that the compensatory increase in NADPH allowed normal levels to be maintained. In addition, the antioxidants, carnosine and homocarnosine, were increased in the knockout cells, and so provided a reduction in oxidative stress. The taurine biosynthesis pathway is also affected by oxLDL treatment and this pathway can provide another mechanism for countering oxidative stress. The exact function of TSPO remains unknown, although it has been associated with cholesterol transport and regulation of oxidative stress, and, therefore, knockout of the gene could promote oxidative stress [13].

Oxidative stress is a hallmark of retinal degeneration despite results from different studies being inconsistent with regard to the association of age-related changes in oxidative markers in RPE cells [22,23]. The major effect of oxLDL on both wildtype and knockout cells is to increase the markers of oxidative stress, including GSSG, phosphogluconate and NADPH. Thus, wildtype cells appear to behave in a similar way to the untreated knockout cells, and in the treated knockout cells, the pathways that indicate oxidative stress are further increased, consistent with early studies which showed that loss of TSPO resulted in increased ROS production in RPE and steroidogenic cells [11,24].

There is an indication that glycolysis is up-regulated in knockout cells with glyceraldehyde 3-phosphate, glucose 6-phosphate, fructose phosphate, fructose bisphosphate and phosphoglyceric acid being increased in knockout cells compared to wildtype cells. In addition, some TCA cycle metabolites, citrate and aconitic acid were also elevated. Linked to this is a slight elevation of ATP and NADH, and a marked increase in creatine phosphate (CPi, Table S1), which is required for exporting ATP derived from the electron transport chain out of the mitochondrion [25,26]. In addition, creatine, the precursor of CPi, and creatinine, its breakdown product, were also elevated. Altogether this suggests that energy metabolism is up-regulated in the knockout cells, possibly in order to counteract the effects of oxidative stress. The glycolysis pathway is believed to generate about 50% of ATP in the retina [27]. Studies have been carried out in vivo to investigate the impact of inducing glycolysis in RPE cells and found that neighboring photoreceptors degenerated as a consequence [26,28]. A less expected effect is that several acyl carnitines were up-regulated by oxLDL treatment in both knockout and wildtype cells. The most marked elevations were in the long chain acyl carnitines, stearoyl and palmitoyl carnitine, and this suggests an increase in fatty acid β-oxidation in order to derive more energy metabolites, and potentially, NADPH. Carnitine conjugation is required in order for the fatty acids to enter the mitochondria. It has been observed that aged retinal cells lose some of their capacity for energy metabolism, which makes them less able to adapt to oxidative stress [29].

As previously established, TSPO deletion deteriorates cholesterol efflux and enhances lipid accumulation in cells [11]. Knockout of TSPO in steroidogenic cells (MA-10 Leydig cell line) also resulted in increased uptake and oxidation of fatty acid [24]. In the current study, lipid metabolism was the most affected pathway, with about 51% of all identified metabolites. There were differences in the lipid composition between the KOC and WTC cells, with the WTC cells having a greater abundance of ether lipids. Ether lipids, with a double bond adjacent to the oxygen to which they are bonded, are known as plasmalogens and have been found to be important in myelination, with glycerolipids in myelin containing up 70% plasmalogens [30]. Amongst the abundant lipids, some are increased and some are decreased by oxLDL treatment, suggesting remodeling of the cell membrane to counteract oxidative stress. There were also marked effects on the lipid composition resulting from oxLDL treatment amongst less abundant lipids. Some lipids were enriched by the treatment and some were depleted. The depleted lipids include some very long chain lipids, such as PG44:12. Such lipids are typically found in the retina where docosahexenoic acid is abundant and where it is incorporated into lipids typically containing two docosahexanoyl chains. Low levels of docosahexenoic acid have been associated with retinitis pigmentosa [31]. Long chain unsaturated fatty acids within lipids are susceptible to oxidative damage and there are four lipids with unsaturation >7 that were depleted in both KOC and WTC cells by oxLDL treatment. Numerous studies have emphasized the influence of lipid modification in RPE cells on the metabolic processes of the retina [32,33,34]. As RPE cells process and recycle the lipids from lipid-rich photoreceptor outer segments (POS) throughout life to maintain visual function, RPE cell lipid disturbance will subsequently affect photoreceptors, leading to further visual dysfunction due to lipid accumulation and lipid peroxidation products [35]. Even though lipid accumulation may not harmfully influence RPE cell function, in combination with oxidative stress over time it could lead to formation of lipid peroxidation products, such as malondialdehyde (MDA) [36]. Lipid progressive accumulation will also stress the RPE, which in turn induces cell apoptosis that is known to be the initial process of AMD disease [37,38]. Particularly, significant dysregulation was mainly found in the glycerophospholipids, the major component of cell membranes, which is enriched in neural membranes. In addition to their importance for providing membrane fluidity and structural stability, glycerophospholipids, along with sphingolipids, appear to play a fundamental role in generating and expanding oxidative stress in neurologic disorders [39]. They were also found to play an essential role in neural cell proliferation, differentiation, and apoptosis. RPE cell membrane impairment due to phospholipid dysregulation emphasizes the importance of the role of lipids in AMD and other neurodegenerative diseases. A study carried out by Suzuki et al. revealed that oxidized phosphatidylcholine levels in the photoreceptors and RPE of the human macular area increased with age [40]. Higher intense immunoreactivity for oxidized phospholipids was noticed among eyes with AMD than in normal eyes. Application of sub-retinal oxidized phospholipids was reported to induce choroidal neovascularization in mice [41]. From the current results, it is difficult to see a clear trend, with some lipids being up-regulated and some being down-regulated. One group of lipids where there is a clear up-regulation is in the sphingolipids. Two ceramides were higher in knockout cells and increased in both knockout and wildtype cells following oxLDL treatment. It has been observed that sphingomyelins are hydrolyzed to ceramides in response to oxidative stress, which then act as mediators of oxidative stress [42]. Observing the differences of lipid metabolites between knockout and wildtype revealed a disturbance of fatty acids, ceramide/sphingosine lipids and glycerophospholipids, and inducing oxidative stress. This is correlated with a metabolomics study carried out on AMD patients, which reported significant changes, especially in plasma lipid metabolism, compared to control subjects [19]. In that study, among 87 significant metabolites that differed between AMD patient samples and controls, most were involved in lipid metabolism, including fatty acids, diacylglycerols, phosphatidylcholines and phosphatidylinositols.

Our observations relate to cell-based work on retinal pigment epithelial cells, which might not be principally relevant to an in vivo setting. Impacts of neighboring cells of photoreceptors, in addition to the inflammatory and immune systems, cannot be taken into account. Accordingly, the metabolic modification described in our RPE cell culture model may vary from an in vivo setting based on the same intervention. Therefore, it is fundamental to examine the current study findings on an animal model, such as a *Tspo* knockout mouse model. In addition, the limited number of authentic standards, which are often not available, restricted our ability to confirm all involved metabolites, particularly lipids. Thus, quite a number of compounds were only identified to MSI level 2. Independent biological replicates would be required to fully confirm the observations made. In further in vivo and in vitro work, it might be useful to look at the effect of added anti-oxidants on the metabolic shifts observed in the current work. In addition, it would important to gain more understanding of the structures of the lipids in these cell lines. The ether lipids seem particularly abundant and this requires confirmation.

## 4. Materials and Methods

### 4.1. Sample Preparation

The *TSPO* knockout ARPE-19 cell line was created in our laboratory [11]. Wildtype and *TSPO* Knockout cells (passage 4) were seeded in a six-well plate (6 × 10^5^ cells/well) and grown for 24 h at 37 °C in an incubator with 5% CO_2_. The cells were treated with oxLDL for 24 h. Afterwards, culture media were removed, and adherent cells were washed with phosphate-buffered saline (PBS) at 37 °C. A cooled (−20 °C) extraction cocktail of methanol/acetonitrile/water at ratio 50:30:20 was used to extract a variety of polar and non-polar putative metabolites. The volume of extraction cocktail was added to each well based on the ratio of 1 mL: 1 × 10^6^ cell. Cells were then scraped, and cell lysates were mixed on a mixer rotor at 1440 rpm for 12 min at 4 °C, before they were centrifuged at 15,300 rpm for 15 min at 0 °C. Supernatants were subjected to further analysis using liquid chromatography mass spectrometry (LC/MS). Each group had samples of (*n* = 3) and the study was done in duplicate. Mixtures of authentic standard metabolites [43] and pooled quality control (QC) samples were injected in order to facilitate identification and to evaluate the sensitivity and reproducibility of the analytical method. Pooled samples (QC) were prepared by obtaining 10 µl from each sample and aliquoted in separate LC/MS analysis vials. It was run frequently throughout the experiment after every three samples. The five readings of pooled (QC) samples provided fundamental evidence for the analysis efficiency. Relative standard deviation (RSD) was calculated based on those readings for each metabolite. The metabolites of RSD < 30% only were included. Authentic standards were run with the samples in the same experiment to help with the confirmation of the metabolite by matching the retention time (RT) of that metabolite in the samples and the standard.

### 4.2. Liquid Chromatography Mass Spectrometry (LC/MS) Analysis

LC/MS grade water and acetonitrile (ACN) purchased from Fisher Scientific and prepared as aqueous mobile phase A (20 mM ammonium carbonate buffer, pH 9.2) and organic mobile phase B was HPLC-grade (acetonitrile) at a flow rate of 300 μL/min and sample injection volume of 10 µl. Based on standard lab procedure, the elution gradient was an A:B ratio of 20:80 at 0 min, 80:20 at 30 min, 92:8 at 35 min and finally 20:80 at 45 min as described previously [43]. Samples were kept in a vial tray that was conditioned at a steady temperature of 4 °C to prevent sample degradation. Mobile phase solutions were newly prepared and stored at room temperature for up to 24 h.

Chromatographic separation was employed on an Accela HPLC system interfaced to an Exactive Orbitrap mass spectrometer (Thermo Fisher Scientific) utilizing a hydrophilic interaction liquid chromatography column (ZICp-HILIC, 150 × 4.6 mm, 5 µm particle size) supplied by Hichrom Ltd. (Reading, UK). Flow rates of the nitrogen sheath and auxiliary gas were sustained at 50 and 17 arbitrary units. Positive negative switching of the electrospray ionisation (ESI) interface was operated with 4.5 kV in positive mode and 4.0 kV in negative mode. The ion transfer capillary temperature was 275 °C. Complete data scans were obtained in the mass-to-charge ratio (*m*/*z*) range of 75 to 1200 for both negative and positive modes with settings of AGC target and resolution as balanced (1 × 10^6^) and high (50,000), respectively. Data were then documented using the Xcalibur 2.1.0 software (Thermo Fisher Scientific).

### 4.3. Data Extraction and Processing

Raw data were placed into different groups based on the intervention and had been converted to (mzXML) files, while polarity was split using the MzMatch split function to discrete the Exactive’s positive and negative ion mass spectra. Split files were run on R using centwave function where peaks were elected and files converted to the format of peakml. Centwave settings were: (<2) ppm mass deviation, (5–100) seconds baseline peak width, signal to noise ratio (3), prefilter intensity and Mzdiff were (1000) and (0.001), respectively. Parameter settings for the MzMatch filters were: mass deviation from sample to another was (5 ppm) and retention time (RT) deviation was (0.5 min). MzMatch filtrations also consider: RSD filter (0.5), where peak reproducibility was measured by the RSD of peak intensities for each group of replicates; noise filter (0.8), while peak shape was evaluated by CoDA-DW score (0–1); intensity filter (1000), where features are removed if no sample had a peak above the intensity threshold; and, detection filter (2), when peaks must be present in a minimum number of two samples of each group. Ultimate lists of peaks were marked with a confidence level of 0 to 10 (10 = most confident) based on the identification of each metabolite; confidence <5 was rejected as false identification. Databases used to identify the biochemicals were IDEOM [44]; KEGG (http://www.genome.jp/kegg/); human metabolome databases (http://www.hmdb.ca/); and Lipid map (http://www.lipidmaps.org/). Where the metabolite did not match the retention time of an authentic standard, it was identified based on elemental composition alone and thus can be considered to be identified to MSI level 2 [45]. Although, IDEOM has an element of retention time prediction based on hydrophilic partitioning built into it [46].

### 4.4. Statistical Analysis

IDEOM of MzMatch software [46] was applied to convert the raw data peaks into numeric values for further processing and analysis. Data were then analyzed using univariate (Excel) and multivariate (SIMCA-P) methods to determine the most significant metabolites and pathways affected by the intervention. Data were log transformed prior to placing them into SIMCA-P multivariate tool before the data were analyzed and filtered finally on specific criteria (see Figure S1 and Table S1). Multivariate analysis SIMCA-P software v.14 (Umetrics AB, Umea, Sweden) was utilized to process and visualize the data. Metaboanalyst 3.0 (www.metaboanalyst.ca) [47] and Microsoft Excel 2010 were employed for univariate analysis. The study used student’s *t*-test p value to examine the significant difference between the groups. Metabolites with a p value < 0.05 were then examined by adjusted *p*-value (FDR) cutoff 0.05 [48].

On the SIMC-P, observations were labelled based on their group and data were Pareto scaled. The data were then fitted based on default settings to create a PCA plot. The differences between groups, if applicable, were investigated using OPLS-DA. SIMCA-P classification by OPLS-DA provided a 95% confidence interval that could be taken from the data description of the OPLS-DA model and variable importance for the projection (VIP) values and these values could be used to filter out the most significant metabolites.

## 5. Conclusions

The most consistent changes in KOC cells and oxLDL treated KOC and WTC cells were with regard to increased glycolysis and an increase in indicators of oxidative stress, such as GSSG, which were increased by either *TSPO* knockout or by treatment with oxLDL. Thus, regardless of the effect that *TSPO* knockout might have on cholesterol transport, the major effect of its absence might be to cause tissue damage via oxidative stress. This is underlined by the fact that the effect of oxLDL is to promote similar metabolic changes in WTC that are observed in the KOC cells without treatment. In the treated KOC cells, oxLDL further promotes the metabolic changes that were produced by the gene knockout. The changes in the lipids in the treated KOC and WTC cells are too variable to propose a clear mechanism. It is evident that there are differences in the phospholipid profiles of the KOC and WTC cells but it is difficult to link these to a disease mechanism because the lipids are both up- and down-regulated by treatment. In contrast, several markers of oxidative stress are clearly up-regulated in KOC and WTC cells. It is clear from the response of both the KOC and WTC cells to oxLDL treatment that both cells are able to counter oxidative stress by maintaining GSH levels and increasing levels of antioxidants such as urate, carnosine and homocarnosine. The cells do this by up-regulating their metabolism particularly with respect to glycolysis, the pentose phosphate pathway and fatty acid β-oxidation. Our findings may shed new light on the disease mechanisms of AMD.

## Supplementary Materials

Supplementary materials can be found at https://www.mdpi.com/1422-0067/20/6/1387/s1.
