# Supplementary material for: Deletion of TSPO Resulted in Change of Metabolomic Profile in Retinal Pigment Epithelial Cells
Source: Int J Mol Sci. 2019 Mar 19;20(6):1387. doi: 10.3390/ijms20061387 (PMC6470938; doi:10.3390/ijms20061387)
Supplement: Supplementary file 1 [file ijms-20-01387-s001.pdf]

# Supplementary Materials

**Table 1.** Comparison of metabolite changes between KOT and KOC, WTT/WTC, KOT/WTT, or KOC/WTC. WTC: Wildtype cells without oxLDL treatment; WTT, Wildtype cells with oxLDL treatment. KOC, *TSPO* knockout cells without oxLDL treatment; KOT, *TSPO* knockout cells with oxLDL treatment.

| Mass (m/z) | RT(min) | Putative Metabolite | +/- | KOT/KOC | | WTT/WTC | | KOT/WTT | | KOC/WTC | |
|---|---|---|---|---|---|---|---|---|---|---|---|
| | | **Arginine/creatine metabolism** | | P value | Ratio | P value | Ratio | P value | Ratio | P value | Ratio |
| 89.05 | 14.3 | sarcosine * | + | 0.003 | 2.75 | 0.001 | 2.58 | 0.012 | 1.55 | 0.098 | 1.45 |
| 113.06 | 15.3 | Creatinine* | - | 0.002 | 2.68 | 0.004 | 3.36 | 0.044 | 1.73 | 0.005 | 2.17 |
| 115.06 | 13.4 | Proline * | - | 0.012 | 1.36 | 0.012 | 1.66 | 0.114 | 1.16 | 0.037 | 1.42 |
| 123.99 | 12.2 | Phosphonoacetaldehyde | + | 0.972 | 0.95 | 0.047 | 0.81 | 0.179 | 0.86 | 0.19 | 0.73 |
| 131.07 | 14.2 | Creatine * | + | 0.007 | 3.39 | <0.001 | 4.69 | 0.001 | 1.74 | 0.033 | 2.41 |
| 174.11 | 26.5 | Arginine * | + | 0.041 | 0.69 | 0.04 | 0.73 | 0.049 | 0.71 | 0.054 | 0.75 |
| 132.05 | 15.0 | Asparagine * | + | 0.002 | 0.65 | 0.014 | 0.64 | 0.046 | 1.27 | 0.055 | 1.25 |
| 189.06 | 14.1 | N-Acetyl-L-glutamate | - | 0.086 | 2.17 | 0.001 | 2.92 | 0.012 | 2.24 | 0.046 | 3.02 |
| 211.04 | 15.1 | Phosphocreatine* | + | <0.001 | 2.9 | <0.001 | 7.43 | 0.002 | 1.37 | <0.001 | 3.5 |
| | | **Histidine/lysine metabolism** | | | | | | | | | |
| 156.05 | 11 | 4-Imidazolone-5-propanoate | + | 0.03 | 1.52 | 0.01 | 1.67 | 0.615 | 0.94 | 0.833 | 1.03 |
| 159.09 | 9.3 | 5-Acetamidopentanoate | + | 0.264 | 0.13 | 0.043 | 0.03 | 0.063 | 0.84 | 0.267 | 0.21 |
| 190.06 | 16.6 | N-Carbamyl-L-glutamate * | - | 0.001 | 1.75 | <0.001 | 1.87 | <0.001 | 1.66 | 0.002 | 1.77 |
| 188.12 | 11.6 | N6-Acetyl-L-lysine * | + | 0.256 | 1.18 | 0.016 | 1.65 | 0.002 | 0.58 | 0.263 | 0.8 |
| | | **Glycolysis** | | | | | | | | | |
| 88.02 | 8.3 | Pyruvate * | - | 0.004 | 0.69 | 0.001 | 0.64 | 0.043 | 1.22 | 0.039 | 1.12 |
| 92.05 | 10.1 | Glycerol | - | 0.287 | 0.14 | 0.035 | 0.07 | 0.426 | 1.04 | 0.418 | 0.47 |
| 168.99 | 16.2 | Glyceraldehyde 3-P* | - | 0.002 | 1.668 | 0.031 | 1.409 | <0.001 | 3.009 | <0.001 | 2.542 |
| 182.08 | 13.6 | Mannitol | - | 0.696 | 0.98 | 0.011 | 1.57 | 0.126 | 1.17 | 0.002 | 1.87 |
| 185.99 | 17.2 | 3-Phospho-D-glycerate | - | 0.03 | 0.76 | 0.004 | 0.81 | 0.008 | 1.37 | 0.005 | 1.45 |
| 260.03 | 16.8 | Glucose 1-phosphate * | - | 0.015 | 1.09 | 0.042 | 1.58 | 0.144 | 1.07 | 0.043 | 1.55 |
| 260.03 | 17.2 | Fructose 6-phosphate * | - | 0.566 | 0.94 | 0.003 | 1.21 | 0.047 | 1.15 | 0.013 | 1.48 |
| 340 | 18.5 | D-Fructose 1,6-bisphosphate* | - | 0.042 | 1.27 | 0.039 | 1.49 | 0.023 | 1.63 | <0.001 | 1.92 |
| | | **TCA cycle** | | | | | | | | | |
| 118.03 | 13.9 | Succinate * | - | 0.029 | 1.24 | 0.18 | 0.81 | 0.002 | 0.67 | 0.003 | 0.44 |
| 132.01 | 16.3 | Oxaloacetate * | - | 0.234 | 1.01 | <0.001 | 1.21 | <0.001 | 0.86 | 0.008 | 1.03 |
| 174.02 | 18.4 | cis-Aconitate* | - | 0.002 | 1.44 | 0.038 | 1.2 | 0.003 | 1.54 | 0.002 | 1.28 |
| 192.03 | 18.5 | Citrate * | - | 0.008 | 0.85 | 0.004 | 0.78 | <0.001 | 1.91 | <0.001 | 1.74 |
| | | **Glutathione homeostasis** | | | | | | | | | |

| | | | | | | | | | | | |
|---|---|---|---|---|---|---|---|---|---|---|---|
| 230.02 | 15.7 | Ribose 5-phosphate * | - | 0.012 | 1.24 | 0.003 | 1.3 | <0.001 | 1.95 | <0.001 | 2.05 |
| 276.02 | 18.0 | 6-Phospho-D-gluconate | - | 0.048 | 1.31 | 0.013 | 1.52 | 0.006 | 1.28 | 0.038 | 1.49 |
| 307.083 | 14.1 | Glutathione* | + | 0.08 | 1.61 | 0.43 | 1.3 | 0.77 | 1.03 | 0.64 | 0.83 |
| 612.63 | 17.3 | Glutathione disulphide * | - | 0.005 | 2.44 | 0.031 | 1.84 | 0.011 | 2.23 | 0.036 | 1.68 |
| 745.09 | 17.3 | NADPH * | + | 0.003 | 1.23 | 0.03 | 1.16 | 0.001 | 1.35 | 0.007 | 1.27 |
| | **Indicators of oxidative stress** | | | | | | | | | | |
| 136.04 | 9.8 | Hypoxanthine * | + | 0.573 | 0.83 | 0.755 | 1.03 | 0.03 | 1.54 | 0.059 | 1.91 |
| 152.03 | 11.2 | Xanthine* | - | 0.003 | 0.59 | 0.002 | 0.53 | 0.004 | 1.57 | 0.019 | 1.43 |
| 155.07 | 14.2 | Histidine * | + | 0.024 | 1.3 | 0.015 | 1.18 | 0.026 | 1.19 | 0.333 | 1.08 |
| 168.03 | 12.6 | Urate* | - | <0.001 | 1.57 | 0.021 | 2.3 | 0.102 | 0.61 | 0.03 | 0.89 |
| 226.106 | 15.2 | carnosine | + | 0.06 | 1.14 | 0.1 | 1.32 | 0.047 | 1.33 | 0.01 | 1.5 |
| 240.122 | 13.6 | homocarnosine | + | 0.04 | 1.22 | 0.28 | 0.88 | 0.011 | 1.85 | 0.01 | 1.34 |
| | **Taurine/cysteine metabolism** | | | | | | | | | | |
| 109.02 | 14.7 | Hypotaurine | + | 0.217 | 1.17 | 0.016 | 1.38 | 0.433 | 0.97 | 0.392 | 1.15 |
| 105.04 | 15.5 | Serine * | - | 0.014 | 0.75 | 0.016 | 0.71 | 0.605 | 0.97 | 0.435 | 0.92 |
| 125.014 | 15.0 | Taurine* | + | 0.44 | 0.95 | 0.62 | 1.02 | 0.019 | 1.17 | 0.03 | 1.26 |
| 125.999 | 11.0 | Hydroxyethansulphonate | - | 0.004 | 1.47 | 0.006 | 1.72 | 0.335 | 0.93 | 0.42 | 1.09 |
| 147.05 | 10.5 | O-Acetyl-L-serine | - | 0.002 | 1.7 | 0.005 | 2.51 | 0.313 | 0.9 | 0.128 | 1.33 |
| 153.01 | 14.4 | 3-Sulfino-L-alanine | - | <0.001 | 2.9 | 0.005 | 2.34 | 0.001 | 1.4 | 0.419 | 1.12 |
| 169 | 15.8 | Cysteic Acid | - | 0.001 | 2.92 | <0.001 | 3.87 | 0.022 | 1.49 | 0.001 | 1.97 |
| | **Pyrimidines and purines** | | | | | | | | | | |
| 111.04 | 11.4 | Cytosine * | + | 0.954 | 0.97 | 0.049 | 0.79 | 0.001 | 0.46 | 0.004 | 0.38 |
| 114.04 | 12.0 | 5,6-Dihydrouracil | + | 0.029 | 0.47 | 0.008 | 0.5 | 0.994 | 1.02 | 0.768 | 1.09 |
| 125.06 | 7.4 | 5-Methylcytosine | + | 0.012 | 0.58 | 0.009 | 0.54 | 0.435 | 0.97 | 0.561 | 0.9 |
| 126.04 | 12.6 | Thymine * | + | <0.001 | 1.91 | 0.011 | 1.4 | <0.001 | 1.48 | 0.332 | 1.08 |
| 128.06 | 14.3 | 5,6-Dihydrothymine | + | <0.001 | 2.38 | 0.001 | 2.59 | 0.001 | 1.76 | 0.003 | 1.91 |
| 146.07 | 14.7 | Glutamine * | + | 0.051 | 0.71 | 0.007 | 0.67 | 0.001 | 1.39 | 0.126 | 1.31 |
| 244.11 | 15.1 | dihydrothymidine | + | 0.016 | 1.6 | 0.013 | 1.65 | 0.051 | 1.26 | 0.142 | 1.3 |
| 268.08 | 10.4 | Inosine | - | 0.001 | 1.2 | 0.291 | 0.95 | 0.022 | 0.9 | 0.001 | 0.72 |
| | **Fatty acids biosynthesis and conjugates** | | | | | | | | | | |
| 102.07 | 12.8 | Pentanoate | + | 0.255 | 0.21 | 0.003 | 0.17 | 0.003 | 0.57 | 0.183 | 0.46 |
| 144.12 | 4.2 | [FA (8:0)] octanoic acid | - | 0.596 | 0.89 | 0.008 | 1.38 | 0.025 | 0.84 | 0.235 | 1.29 |
| 146.09 | 4.5 | hydroxy-heptanoic acid | - | 0.077 | 1.49 | 0.002 | 1.52 | 0.739 | 1.04 | 0.853 | 1.06 |
| 174.13 | 7.3 | hydroxy-nonanoic acid | - | 0.294 | 1.17 | 0.02 | 1.49 | 0.106 | 0.77 | 0.857 | 0.98 |
| 224.14 | 3.8 | [FA] Methyl jasmonate | - | 0.008 | 2.24 | 0.001 | 1.91 | 0.062 | 1.45 | 0.159 | 1.23 |
| 228.14 | 4.2 | Traumatic acid | - | 0.004 | 1.54 | 0.033 | 1.25 | 0.172 | 1.1 | 0.294 | 0.9 |
| 228.17 | 3.9 | oxo-tridecanoic acid | - | 0.307 | 1.22 | 0.028 | 0.74 | 0.078 | 1.43 | 0.263 | 0.86 |

| | | | | | | | | | | | |
|---|---|---|---|---|---|---|---|---|---|---|---|
| 240.17 | 4.0 | oxo-Tetradecenoic acid | - | 0.002 | 5.85 | 0.003 | 4.76 | 0.256 | 1.24 | 0.888 | 1.01 |
| 242.19 | 3.8 | oxo-tetradecanoic acid | - | 0.014 | 1.7 | 0.014 | 1.69 | 0.009 | 1.26 | 0.27 | 1.25 |
| 246.15 | 4.6 | 3-Hydroxydodecanedioicacid | - | <0.001 | 6.37 | 0.001 | 4.76 | 0.145 | 1.37 | 0.721 | 1.03 |
| 278.22 | 3.5 | octadecatrienoic acid | + | <0.001 | 4.73 | 0.032 | 3.03 | 0.077 | 0.67 | 0.092 | 0.43 |
| 284.27 | 3.6 | Octadecanoic acid | - | 0.347 | 1.15 | 0.041 | 0.66 | 0.016 | 1.97 | 0.302 | 1.12 |
| 308.27 | 3.6 | Eicosadienoic acid | - | 0.853 | 1.02 | 0.007 | 0.83 | 0.787 | 0.98 | 0.107 | 0.8 |
| 312.23 | 3.8 | 13S-hydroperoxy-9Z,11E-octadecadienoic acid | - | 0.005 | 3.55 | 0.015 | 3.08 | 0.514 | 1.11 | 0.992 | 0.96 |
| 312.27 | 3.5 | oxo-nonadecanoic acid | + | 0.011 | 1.69 | 0.005 | 2.04 | 0.081 | 1.37 | 0.009 | 1.66 |
| 328.24 | 3.6 | Docosahexaenoic acid | - | 0.492 | 0.91 | 0.018 | 0.69 | 0.015 | 1.68 | 0.08 | 1.27 |
| 330.24 | 4.1 | trihydroxyoctadecenoic acid | - | <0.001 | 26 | <0.001 | 13.58 | 0.014 | 1.47 | 0.356 | 0.77 |
| 340.3 | 3.5 | oxo-heneicosanoic acid | + | 0.005 | 2.26 | 0.003 | 2.39 | 0.032 | 1.4 | 0.085 | 1.48 |
| 368.22 | 4.2 | Prostaglandin G2 | - | <0.001 | >1000 | <0.001 | >1000 | 0.039 | 1.25 | ND | 1 |
| | | **Carnitines** | | | | | | | | | |
| 203.12 | 10.2 | O-Acetylcarnitine* | + | 0.029 | 1.3 | 0.028 | 1.42 | 0.647 | 1.04 | 0.263 | 1.14 |
| 231.15 | 7.9 | O-Butanoylcarnitine | + | 0.011 | 0.12 | 0.001 | 0.19 | 0.005 | 0.32 | 0.125 | 0.5 |
| 399.33 | 4.2 | L-Palmitoylcarnitine | + | <0.001 | 7.4 | 0.002 | 12.62 | 0.348 | 0.69 | 0.449 | 1.19 |
| 425.35 | 4.2 | Oleoylcarnitine | + | 0.004 | 2.26 | 0.012 | 2.78 | 0.648 | 0.89 | 0.547 | 1.09 |
| 427.37 | 4.1 | Stearoylcarnitine | + | 0.001 | 5.57 | 0.001 | 7.96 | 0.6 | 0.87 | 0.446 | 1.24 |
| | | **Energy metabolism** | | | | | | | | | |
| 347.06 | 13.4 | AMP* | + | <0.001 | 1.46 | <0.001 | 1.49 | 0.154 | 1.05 | 0.123 | 1.07 |
| 427.03 | 15.1 | ADP * | - | 0.237 | 1.1 | 0.026 | 1.23 | 0.102 | 1.18 | 0.002 | 1.33 |
| 443.02 | 18.2 | GDP * | - | 0.075 | 1.33 | 0.018 | 1.81 | 0.748 | 1.03 | 0.047 | 1.41 |
| 507 | 16.7 | ATP * | - | 0.006 | 1.18 | 0.008 | 1.34 | 0.145 | 1.1 | 0.004 | 1.25 |
| 522.99 | 19.8 | GTP* | - | 0.05 | 1.28 | 0.08 | 1.3 | 0.13 | 1.11 | 0.43 | 1.12 |
| 665.12 | 13.1 | NADH * | - | 0.005 | 1.41 | 0.004 | 1.6 | 0.383 | 1.08 | 0.018 | 1.23 |
| | | **Sphingolipids** | | | | | | | | | |
| 273.27 | 5.2 | Hexadecasphinganine | + | <0.001 | 23.71 | <0.001 | 35.73 | 0.197 | 0.76 | 0.403 | 1.15 |
| 299.28 | 7.3 | Sphingenine | + | 0.372 | 0.78 | <0.001 | 0.8 | 0.001 | 1.75 | 0.053 | 1.78 |
| 327.31 | 4.0 | N,N-Dimethylsphing-4-enine | + | 0.006 | 2.65 | 0.003 | 2.34 | 0.613 | 0.92 | 0.22 | 0.81 |
| 329.33 | 4.8 | dimethylaminooctadecanediol | + | 0.003 | 2.15 | 0.002 | 1.91 | 0.016 | 1.62 | 0.016 | 1.44 |
| 535.5 | 3.7 | Cer (d18:2/16:0) | - | 0.091 | 1.4 | 0.004 | 2.5 | 0.139 | 0.71 | 0.138 | 1.26 |
| 537.51 | 3.6 | Cer (18:1 16:0) | + | 0.204 | 1.25 | 0.002 | 1.78 | 0.002 | 0.56 | 0.192 | 0.79 |
| 699.57 | 3.7 | Glucosylceramide (d18:1/16:0) | - | 0.585 | 1.12 | 0.01 | 1.22 | 0.628 | 0.94 | 0.69 | 1.03 |
| 730.6 | 3.8 | SM(d18:1/18:0) | + | 0.493 | 1.1 | 0.048 | 1.36 | 0.114 | 0.78 | 0.738 | 0.97 |
| 809.67 | 3.7 | Glucosylceramide (d18:1/24:1) | - | 0.024 | 0.69 | 0.012 | 0.61 | 0.428 | 0.93 | 0.207 | 0.83 |
| | | **Glycerophospholipids** | | | | | | | | | |
| 105.08 | 19 | diethanolamine | + | 0.376 | 0.76 | 0.044 | 0.5 | 0.569 | 0.85 | 0.056 | 0.56 |

| | | | | | | | | | | | |
|---|---|---|---|---|---|---|---|---|---|---|---|
| 410.24 | 4.3 | LPA 16:0 | - | 0.002 | 2.25 | 0.031 | 2.04 | 0.018 | 1.61 | 0.134 | 1.46 |
| 437.29 | 4.2 | LPE 16:0 ether | - | 0.002 | 2.06 | 0.005 | 1.97 | 0.001 | 2 | 0.009 | 1.91 |
| 438.27 | 4.1 | LPA 18:0 | - | 0.001 | 2.74 | 0.014 | 2.31 | 0.015 | 1.31 | 0.588 | 1.1 |
| 453.29 | 4.3 | LPE 16:0 | + | 0.106 | 2.05 | <0.001 | 2.89 | <0.001 | 1.53 | 0.205 | 2.16 |
| 481.35 | 4.3 | Lyso PC 16:2 | + | 0.194 | 8.27 | <0.001 | 10.21 | 0.177 | 0.82 | 0.436 | 1.02 |
| 495.33 | 4.3 | LPC 16:0 | + | 0.059 | 2.23 | 0.002 | 3.65 | 0.026 | 1.4 | 0.112 | 2.29 |
| 509.38 | 4.2 | Lyso PC 18:0 ether | + | 0.195 | 7.88 | <0.001 | 5.04 | 0.03 | 0.62 | 0.307 | 0.39 |
| 523.36 | 4.2 | LPC 18:0 | + | 0.005 | 3.74 | 0.001 | 4.92 | 0.573 | 1.14 | 0.172 | 1.5 |
| 676.54 | 3.7 | PA O-18:0/17:0 | + | <0.001 | 5.7 | 0.002 | 5.19 | 0.761 | 1.03 | 0.886 | 0.94 |
| 678.48 | 3.5 | LPG 30:1 ether | - | 0.001 | 2.64 | 0.01 | 2.17 | 0.111 | 1.41 | 0.154 | 1.16 |
| 680.5 | 3.5 | PG 30:0 ether | - | <0.001 | 4.38 | 0.008 | 2.4 | 0.213 | 1.26 | 0.039 | 0.69 |
| 694.48 | 3.5 | PG 30:0 | - | 0.002 | 0.46 | 0.013 | 0.54 | 0.008 | 1.87 | 0.003 | 2.22 |
| 720.49 | 3.5 | PG 32:1 | - | 0.008 | 0.56 | 0.03 | 0.65 | 0.013 | 1.72 | 0.005 | 2 |
| 722.51 | 3.5 | PG 32:0 | - | 0.003 | 0.49 | 0.004 | 0.64 | 0.001 | 1.79 | 0.002 | 2.32 |
| 734.55 | 3.5 | LPG 34:1 ether | - | <0.001 | 2.38 | 0.025 | 1.66 | 0.56 | 1.06 | 0.084 | 0.74 |
| 771.54 | 3.8 | PS 36:3 ether | - | 0.05 | 34.24 | 0.12 | >1000 | 0.32 | 2.52 | 0.37 | >1000 |
| 773.54 | 3.6 | PE 40:7 ether | - | 0.017 | 0.75 | 0.001 | 0.64 | 0.538 | 1.06 | 0.068 | 0.9 |
| 773.557 | 3.7 | PS 36:2 ether | + | 0.06 | 139.3 | 0.12 | 19.83 | 0.19 | 1.05 | 0.85 | 0.15 |
| 776.56 | 3.5 | PG 36:1 | - | 0.071 | 2.07 | 0.011 | 1.79 | <0.001 | 1.23 | 0.965 | 1.07 |
| 783.58 | 3.7 | PC 36:3 | + | <0.001 | 2.25 | <0.001 | 2.29 | 0.145 | 1.12 | 0.001 | 1.14 |
| 785.52 | 3.5 | PS 36:3 | - | <0.001 | 2.44 | 0.002 | 3.01 | 0.07 | 0.78 | 0.855 | 0.96 |
| 797.56 | 3.7 | PS 38:4 ether | + | <0.001 | >1000 | <0.001 | >1000 | 0.014 | 1.14 | *ND | 1 |
| 799.57 | 3.7 | PS 38:3 ether | + | <0.001 | 9.45 | <0.001 | 9.86 | 0.355 | 1.08 | 0.477 | 1.13 |
| 815.57 | 3.5 | PS 38:2 | - | 0.01 | 0.66 | 0.035 | 0.79 | 0.052 | 0.77 | 0.277 | 0.92 |
| 817.58 | 3.5 | PS 38:1 | - | 0.042 | 0.74 | 0.003 | 0.74 | 0.025 | 0.81 | 0.08 | 0.81 |
| 831.58 | 3.7 | PC 40:7 | + | 0.001 | 0.47 | 0.05 | 0.61 | 0.181 | 1.2 | 0.037 | 1.56 |
| 843.6 | 3.5 | PS 40:2 | - | 0.003 | 0.52 | 0.001 | 0.62 | 0.017 | 0.67 | 0.027 | 0.81 |
| 858.53 | 3.5 | PI 36:4 | - | 0.002 | 2.24 | <0.001 | 2.41 | 0.008 | 1.36 | 0.037 | 1.46 |
| 859.61 | 3.7 | PC 42:7 | + | 0.015 | 0.55 | 0.007 | 0.52 | 0.1 | 1.22 | 0.44 | 1.16 |
| 866.51 | 3.4 | PG 44:12 | - | 0.003 | 0.54 | 0.014 | 0.66 | 0.005 | 1.51 | 0.006 | 1.84 |
| 871.63 | 3.5 | PS 42:2 | - | 0.001 | 0.3 | <0.001 | 0.4 | <0.001 | 0.6 | 0.127 | 0.81 |
| 879.59 | 3.6 | PI 36:0 | + | 0.157 | 0.57 | 0.038 | 0.65 | 0.001 | 0.4 | 0.045 | 0.45 |
| 900.57 | 3.5 | PI 17:0/22:4 | - | 0.013 | 0.77 | 0.003 | 0.7 | 0.164 | 1.1 | 0.895 | 1.01 |
| | | **Miscellaneous** | | | | | | | | | |
| 100.05 | 4.4 | glutaraldehyde | - | 0.839 | 1.01 | 0.002 | 0.66 | <0.001 | 1.77 | 0.336 | 1.15 |
| 172.01 | 13.8 | sn-glycerol-1-phosphate | - | 0.088 | 1.52 | 0.003 | 2.22 | 0.08 | 1.29 | 0.04 | 1.88 |
| 179.08 | 10.7 | D-Glucosamine or isomer | - | 0.634 | 1.11 | 0.043 | 1.42 | 0.723 | 0.96 | 0.152 | 1.23 |

| | | | | | | | | | | | |
|---|---|---|---|---|---|---|---|---|---|---|---|
| 187.1 | 13.3 | 5-guanidino-3-methyl-2-oxo-pentanoate | + | 0.007 | 0.46 | 0.031 | 0.5 | <0.001 | 3.02 | 0.008 | 3.3 |
| 466.311 | 3.5 | Cholesterolsulfate | - | <0.001 | 3.47 | <0.001 | 3.49 | 0.82 | 1.04 | 0.75 | 1.05 |

*ND: not detected at control samples without oxLDL treatment means there was no sample peak or it was below the range of detection.; m/z: the mass-to-charge ratios; RT: metabolite matches standard retention time.

| | A | B | C | D | E | F | G | H | I | J | K | L | M | N | O | P | Q | R | S | T | U | V | W |
|---|---|---|---|---|---|---|---|---|---|---|---|---|---|---|---|---|---|---|---|---|---|---|---|
| 1 | Id | Mass (m/z | RT | Putative m | Map | Pathway | K1C1 | K1C2 | K1C3 | K1T1 | K1T2 | K1T3 | W1C1 | W1C2 | W1C3 | W1T1 | W1T2 | W1T3 | P1 | P2 | P3 | P4 | P5 |
| 2 | 116 | 174.0165 | 18.4534 | cis-Aconita | Carbohydr | Citrate cyc | 277201.8 | 285804.9 | 283967 | 370951.5 | 436722.6 | 413303.9 | 205898.1 | 229285 | 224589.6 | 286089.5 | 263709 | 241328.5 | 123973.7 | 198451.4 | 161504 | 178796.9 | 152199.5 |
| 3 | 253 | 506.9956 | 16.70135 | ATP | Energy Me | Oxidative | | 296088.1 | 312551.1 | 298400.8 | 341055 | 372805.2 | 354471.6 | 258816.7 | 233439.3 | 232178.5 | 349988.2 | 323688.1 | 297388 | 364180.5 | 310809.2 | 330553.7 | 353647.1 | 305366 |
| 4 | 335 | 260.0297 | 16.80892 | D-Glucose | Carbohydr | Starch anc | 18810.05 | 18719.88 | 17747.04 | 19741.98 | 19994.38 | 20286.43 | 14562.92 | 8555.221 | 12477.78 | 17551.41 | 18727.03 | 19902.64 | 24062.64 | 21674.79 | 20890.02 | 20696.19 | 27204.21 |
| 5 | 365 | 665.1243 | 13.11633 | NADH | Energy Me | Oxidative | | 15141.91 | 13728.67 | 13628.24 | 19929.97 | 18218.88 | 21863.52 | 10585.34 | 11848.03 | 12091.39 | 20519.37 | 18461.3 | 16403.22 | 9053.478 | 16026.06 | 17586.85 | 16599.28 | 21832.55 |
| 6 | 368 | 132.006 | 16.38615 | Oxaloacet | Carbohydr | Citrate cyc | 3401.215 | 3360.957 | 3394.325 | 3403.987 | 3408.014 | 3402.365 | 3318.959 | 3289.92 | 3269.056 | 3959.579 | 3979.408 | 3999.237 | 4017.974 | 2871.003 | 3024.384 | 4332.986 | 3352.996 |
| 7 | 425 | 276.0247 | 18.0255 | 6-Phospho | Carbohydr | Pentose pl | 8244.068 | 9435.379 | 6932.648 | 11474.36 | 10039.91 | 10647.94 | 4806.647 | 5109.573 | 6591.383 | 8745.063 | 8375.714 | 8006.364 | 9362.08 | 9118.036 | 8947.48 | 7307.172 | 7273.222 |
| 8 | 580 | 339.996 | 18.57917 | Fructose 1 | Energy Me | Carbon fix | 6304.875 | 6381.881 | 5973.122 | 6917.875 | 8990.26 | 7736.314 | 3583.249 | 2942.558 | 3169.924 | 5775.322 | 4821.423 | 3867.524 | 11740.59 | 7481.311 | 6543.671 | 7174.382 | 5577.122 |
| 9 | 592 | 192.0273 | 18.55615 | Citrate | Carbohydr | Citrate cyc | 1437440 | 1334940 | 1324176 | 1125156 | 1179010 | 1195760 | 767843 | 810610 | 777958 | 651349 | 609372 | 567395 | 429664.5 | 464463.3 | 313529 | 386802.1 | 333173.5 |
| 10 | 671 | 260.0297 | 15.95649 | Fructose 6 | Carbohydr | Pentose pl | 20810.05 | 25917.74 | 19473.08 | 21983.24 | 21069.3 | 18894.01 | 14562.92 | 14476.57 | 15710.65 | 17551.41 | 18008.79 | 18466.16 | 16114.53 | 18158.54 | 17926.94 | 18586.49 | 20616.17 |
| 11 | 694 | 182.0791 | 13.65299 | Mannitol | Carbohydr | Fructose a | 112161.2 | 111846.5 | 107914 | 112128.9 | 116537.5 | 95994.21 | 52029.36 | 56489.17 | 68921.07 | 83880.36 | 92603.25 | 101326.1 | 52966.14 | 84399.1 | 85662.32 | 100817.2 | 91432.36 |
| 12 | 807 | 118.0267 | 13.93982 | Succinate | Carbohydr | Citrate cyc | 337875.3 | 324929.6 | 274233 | 382493.7 | 379868.2 | 401766.8 | 610007.6 | 882299.7 | 655250.8 | 630796.3 | 578967.2 | 527138.1 | 540805.5 | 378890.1 | 318994.4 | 202570.4 | 127094.3 |
| 13 | 847 | 230.019 | 15.76523 | Ribose 5-p | Carbohydr | Pentose pl | 4863.99 | 5267.79 | 4745.44 | 5730.64 | 6135.31 | 6526.24 | 2302.17 | 2340.55 | 2606.62 | 3159.78 | 3140.21 | 3120.64 | 4759.002 | 5799.389 | 5913.166 | 4679.298 | 5131.037 |
| 14 | 866 | 176.032 | 15.68773 | Ascorbate | Carbohydr | Ascorbate | 23802.15 | 23981.37 | 17264.16 | 29687.16 | 16655.09 | 21687.72 | 14850.43 | 29952.13 | 19013.32 | 26239.97 | 18819.73 | 11399.5 | 26215.92 | 23885.51 | 19208.66 | 12110.71 | 9578.763 |
| 15 | 873 | 185.9929 | 17.27585 | 3-Phospho | Carbohydr | Glycolysis , | 7750.001 | 6335.694 | 7128.477 | 5583.613 | 4740.883 | 5751.232 | 4751.32 | 5149.231 | 4685.215 | 3774.965 | 3918.159 | 4061.352 | 5610.958 | 5058.91 | 4108.764 | 5382.553 | 4926.717 |
| 16 | 885 | 427.0297 | 15.12579 | ADP | Energy Me | Oxidative | | 20165.82 | 19232.19 | 18319.81 | 18923.05 | 23086.82 | 21235.02 | 15337.52 | 13966.98 | 14119.01 | 19486.76 | 17862.45 | 16238.13 | 18079.41 | 22021.4 | 20353.9 | 21149.62 | 20438.88 |
| 17 | 960 | 745.091 | 17.3333 | NADPH | Energy Me | Photosyntl | 6880 | 6530 | 6960 | 8760 | 8000 | 8340 | 5080 | 5140 | 5810 | 6420 | 6070 | 6136.099 | 5659.922 | 6364.469 | 2303.579 | 1097.513 | 772.5307 |
| 18 | 1000 | 88.01604 | 8.295672 | Pyruvate | Carbohydr | Glycolysis , | 656931.6 | 625458.1 | 632009.3 | 392505.7 | 467884.1 | 469707.3 | 529035.8 | 585567.8 | 590899.3 | 383695.3 | 362430.8 | 341166.3 | 233989.9 | 340903.8 | 323120.9 | 340932.4 | 335834.5 |
| 19 | 1116 | 92.04731 | 10.1335 | Glycerol | Carbohydr | Galactose | 1194225 | 131078 | 54589.05 | 64313.63 | 66605.93 | 67614.21 | 351417.2 | 2332150 | 238493.1 | 68310.77 | 63742.87 | 59174.96 | 75691.74 | 175354 | 171877.4 | 201661.6 | 161495.1 |
| 20 | | | | | | | | | | | | | | | | | | | | | | | |
| 21 | m/z: the mass-to-charge ratios | | | ; RT: metabolite matches standard retention time | | | | | | | | | | | | | | | | | | | |

**Figure 1.** Some raw data were used for PCA analysis.

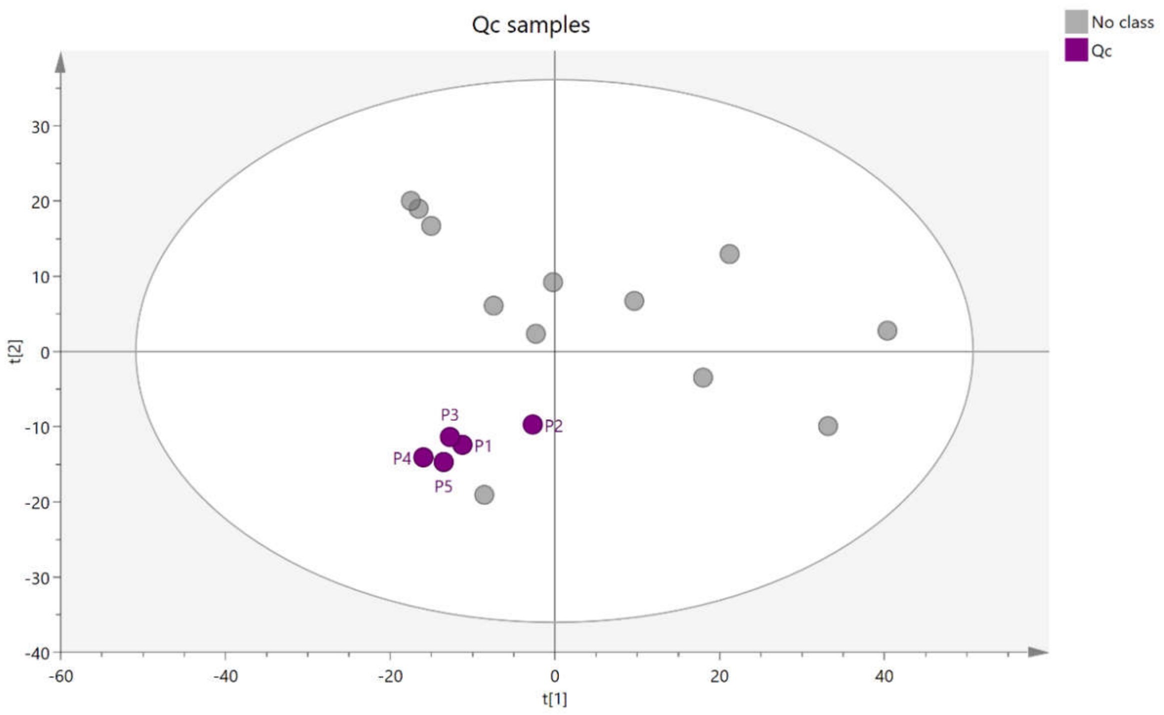


**Figure 2.** Principle component analysis (PCA) score plot for quality control (QC) (pooled) cell extract samples of wildtype and TSPO knockout RPE cells. P1, P2, P3, P4 and P5 are pooled (QC) sample running throughout the experiment and reflecting the goodness of device sensitivity.

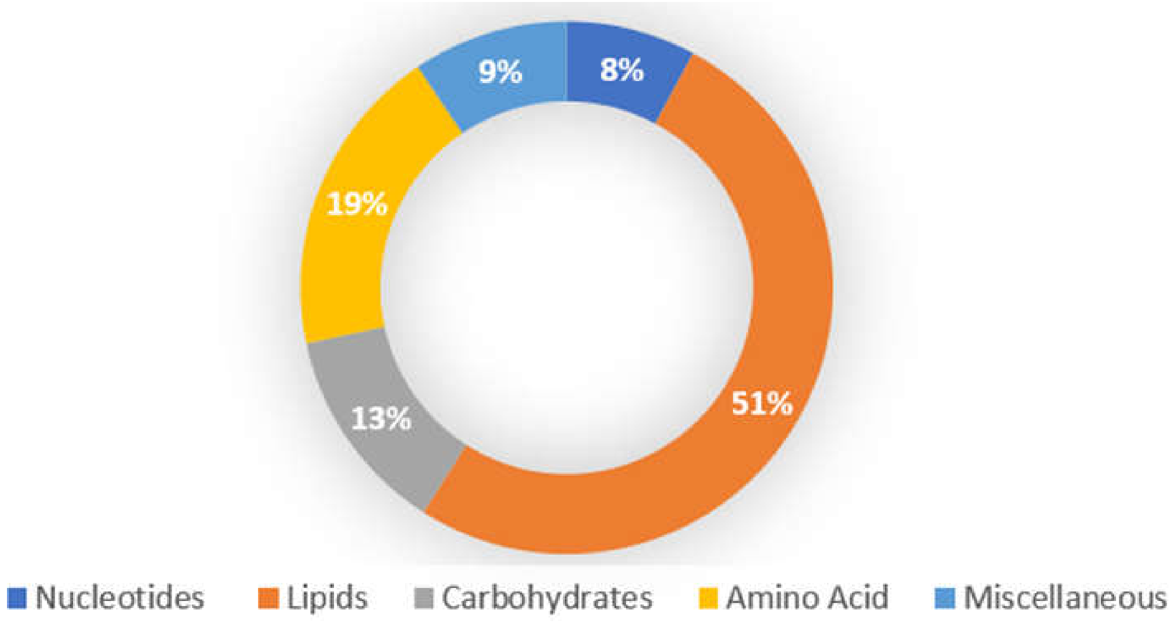


**Figure 3.** Overveiw for implication of different pathways to all significant affected metabolites. Lipid pathway is the most influenced pathway while nucleotides were the least.
